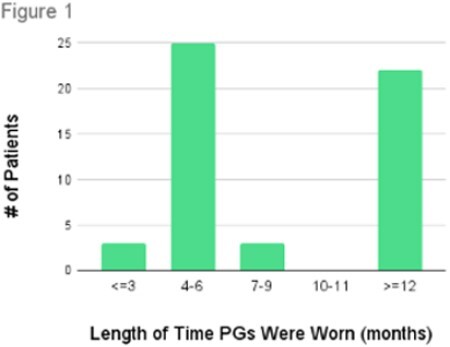# 592 Pressure Garment Use by International Pediatric Patients During the COVID-19 Pandemic: A QI Study

**DOI:** 10.1093/jbcr/irae036.226

**Published:** 2024-04-17

**Authors:** Debbie Minter, Lauren Freeman, David G Greenhalgh, Tina L Palmieri, Kathleen S Romanowski, Soman Sen, Jason Heard

**Affiliations:** Shriners Children's Hospital Northern California, Carmichael, CA; Shriners Children's Northern California, Rancho Cordova, CA; Shriners Hospital Northern California, Sacramento, CA; UC Davis, Shriners Children's Northern California, Sacramento, CA; UC Davis, Sacramento, CA; University of California - Davis Hosp, Sacramento, CA; Shriners Children's Hospital Northern California, Carmichael, CA; Shriners Children's Northern California, Rancho Cordova, CA; Shriners Hospital Northern California, Sacramento, CA; UC Davis, Shriners Children's Northern California, Sacramento, CA; UC Davis, Sacramento, CA; University of California - Davis Hosp, Sacramento, CA; Shriners Children's Hospital Northern California, Carmichael, CA; Shriners Children's Northern California, Rancho Cordova, CA; Shriners Hospital Northern California, Sacramento, CA; UC Davis, Shriners Children's Northern California, Sacramento, CA; UC Davis, Sacramento, CA; University of California - Davis Hosp, Sacramento, CA; Shriners Children's Hospital Northern California, Carmichael, CA; Shriners Children's Northern California, Rancho Cordova, CA; Shriners Hospital Northern California, Sacramento, CA; UC Davis, Shriners Children's Northern California, Sacramento, CA; UC Davis, Sacramento, CA; University of California - Davis Hosp, Sacramento, CA; Shriners Children's Hospital Northern California, Carmichael, CA; Shriners Children's Northern California, Rancho Cordova, CA; Shriners Hospital Northern California, Sacramento, CA; UC Davis, Shriners Children's Northern California, Sacramento, CA; UC Davis, Sacramento, CA; University of California - Davis Hosp, Sacramento, CA; Shriners Children's Hospital Northern California, Carmichael, CA; Shriners Children's Northern California, Rancho Cordova, CA; Shriners Hospital Northern California, Sacramento, CA; UC Davis, Shriners Children's Northern California, Sacramento, CA; UC Davis, Sacramento, CA; University of California - Davis Hosp, Sacramento, CA; Shriners Children's Hospital Northern California, Carmichael, CA; Shriners Children's Northern California, Rancho Cordova, CA; Shriners Hospital Northern California, Sacramento, CA; UC Davis, Shriners Children's Northern California, Sacramento, CA; UC Davis, Sacramento, CA; University of California - Davis Hosp, Sacramento, CA

## Abstract

**Introduction:**

Pressure therapy is a standard intervention for the treatment of hypertrophic scars. Due to the COVID-19 pandemic, our international burn outreach programs were canceled between March 2020 and June 2022 so the ability to monitor pressure garments was lost. During this time, patients were discharged from our burn unit with two sets of properly fitting pressure garments (PGs) but were unable to be provided with replacement sets in their home country. Our burn team used this unique opportunity to perform a QI study of how this lack of access to follow up care impacted our patients’ pressure therapy programs.

**Methods:**

Surveys were given to pediatric patients in three of our reinstated international burn outreach clinics in 2023. Surveys were in the patient's native language with written instructions and were completed by the caregivers while waiting for their appointment. Patients with a date of injury occurring in 2019 or later who were discharged with PGs to their home country from our facility were included in the survey. Patients who were still using PGs were excluded.

**Results:**

Fifty-four patients were included in the study with mean age at time of discharge of 6yr3mo and age range 10mo-17yr. The length of time that PGs were worn is summarized in Figure 1 with 46% of patients stopping PG use between 4-6 months and 39% stopping at one year or greater. The reasons for terminating PG use are summarized in Figure 2 with 68% of patients stopping due to outgrowing the garments. In terms of alternate solutions used, only 9% of patients tried to alter or repair the PGs or sought inexpensive off-the-shelf products as replacements. 17% used bandages when the PGs no longer fit.

**Conclusions:**

A significant number of patients in our outreach clinics stopped using PGs between 4-6 months. This is considerably shorter than the one-year standard of care received by our local patients. A high number of patients also reported wearing them for a year, however, it is questionable how effective the initial sets of PGs would be after this length of time given that evidence suggests garments have significant pressure loss within the first two months. More studies are needed to determine the impact of reduced garment wear on these patients.

**Applicability of Research to Practice:**

Compliance to pressure therapy programs is impacted by access to care. In order to follow best practice guidelines, solutions need to be explored to improve availability of serial sets of properly fitting pressure garments. Additionally, families may benefit from education that empowers them to make alterations and repairs as needed and to seek inexpensive off-the-shelf options when the custom PGs no longer fit.